# Virtual Reality for Enhanced Ecological Validity and Experimental Control in the Clinical, Affective and Social Neurosciences

**DOI:** 10.3389/fnhum.2015.00660

**Published:** 2015-12-11

**Authors:** Thomas D. Parsons

**Affiliations:** Computational Neuropsychology and Simulation Lab, Department of Psychology, University of North TexasDenton, TX, USA

**Keywords:** neuropsychological tests, social neuroscience, ecological validity, virtual reality, neuropsychology

## Abstract

An essential tension can be found between researchers interested in ecological validity and those concerned with maintaining experimental control. Research in the human neurosciences often involves the use of simple and static stimuli lacking many of the potentially important aspects of real world activities and interactions. While this research is valuable, there is a growing interest in the human neurosciences to use cues about target states in the real world via multimodal scenarios that involve visual, semantic, and prosodic information. These scenarios should include dynamic stimuli presented concurrently or serially in a manner that allows researchers to assess the integrative processes carried out by perceivers over time. Furthermore, there is growing interest in contextually embedded stimuli that can constrain participant interpretations of cues about a target’s internal states. Virtual reality environments proffer assessment paradigms that combine the experimental control of laboratory measures with emotionally engaging background narratives to enhance affective experience and social interactions. The present review highlights the potential of virtual reality environments for enhanced ecological validity in the clinical, affective, and social neurosciences.

## Introduction

The issue of ecological validity in psychological assessment has been expressed a number of times over the years via discussions of the limitations of generalizing sterile laboratory findings to the processes normally occurring in people’s everyday lives. In 1978, Neisser presented an opening address at the first International Conference on Practical Aspects of Memory, in which he contended that the experiments found in cognitive psychology were conducted in artificial settings and employed measures that have few counterparts in everyday life. In Neisser’s view such research lacks ecological validity and fails to generalize beyond constrained laboratory settings. In Banaji and Crowder (1989) countered Neisser’s arguments with the claim that the ecological approach lacks the internal validity and experimental control needed for scientific progress. This exchange resulted in a schism between researchers striving for ecological validity and those interested in maintaining experimental control. A great deal of controversy resulted over whether cognitive assessments should emphasize laboratory-based control or more naturalistic approaches (Neisser, 1982; Conway, 1991). A special issue of the American Psychologist (1991) was devoted to this debate. Although the debate over which was superior for cognitive psychology eventually subsided (de Wall et al., 1994), the search for a balance of everyday activities and laboratory control has a long history in clinical neuroscience (Wilson, 1993; Franzen and Wilhelm, 1996; Sbordone and Long, 1996; Chaytor and Schmitter-Edgecombe, 2003; Burgess et al., 2006; Chaytor et al., 2006; Sbordone, 2008) and has most recently been discussed in the affective and social neurosciences (Ochsner, 2004; Schilbach et al., 2006, 2013; Chakrabarti, 2013).

One methodology that has potential for a laboratory vs. everyday functioning rapprochement is virtual reality. Virtual reality makes use of virtual environments to present digitally recreated real world activities to participants via immersive (head-mounted displays) and non-immersive (2D computer screens) mediums. Recent advances in virtual reality technology allow for enhanced computational capacities for administration efficiency, stimulus presentation, automated logging of responses, and data analytic processing. Since virtual environments provide experimental control and dynamic presentation of stimuli in ecologically valid scenarios, they allow for controlled presentations of emotionally engaging background narratives to enhance affective experience and social interactions (Gorini et al., 2011; Diemer et al., 2015).

## Overview and Plan for The Paper

This review aims to describe the potential of virtual reality for precise presentation and control of dynamic perceptual stimuli that can be used for assessment of neurocognitive and affective processing while participants are immersed in simulations of real world contexts. A number of studies in the context of clinical, affective, and social neuroscience research are discussed. While neuroscientific research is highlighted throughout, many of the examples provided reflect assessments of behavioral performance. Although it can be challenging to operationalize the extent to which a stimulus approximates activities of daily living, this review gives examples of stimuli and virtual environment-based contexts that span the implied continuum. This review is not meant to be exhaustive. Instead this review focuses upon research that highlights the ways in which persons respond to clinical, affective, and social stimuli in simulations that approximate real world activities and interactions. The plan for this paper will be as follows: Current Needs for Ecologically Valid Assessments in the Clinical, Affective and Social Neuroscience “Section” discusses current needs for ecologically valid assessments in the clinical, affective, and social neuroscience. For each of these three areas there will be a discussion of current methods and the potential limitations of these approaches for ecologically valid assessments. In Virtual Reality for Enhanced Ecological Validity and Experimental Control “Section”, virtual environments are introduced as methodologies for enhanced ecological validity and experimental control. The potential of virtual environments for clinical, affective, and social neurosciences is presented. Limitations of virtual environments are presented. The manuscript ends with a brief summary and discussion of the ways in which virtual environments can be used for studying how persons respond to clinical, affective, and social stimuli in simulations that approximate real world activities and interactions.

### Current Needs for Ecologically Valid Assessments in the Clinical, Affective and Social Neuroscience

#### Clinical Neuroscience and the Ecological Validity Discussion

The everyday vs. laboratory discussion has continued for the clinical neuroscience specialty of clinical neuropsychology. Much of the impetus for the continued discussion came from a shift in praxes from the “deficit measurement paradigm” to a new paradigm emphasizing functional competence (Chelune and Moehle, 1986). With the advent and development of neuroimaging there was decreased requirement for neuropsychological assessments to localize lesions and an increased need for neuropsychologists to describe behavioral manifestations of neurologic disorders. Franzen and Wilhelm (1996) refined the definition of ecological validity for neuropsychological assessment via an emphasis upon two requirements: (1) Veridicality, in which the patient’s performance on a construct-driven measure should predict some feature(s) of the patient’s day-to-day functioning (e.g., vocational status; Bayless et al., 1989; Dunn et al., 1990; Lysaker et al., 1995) and (2) Verisimilitude, in which the requirements of a neuropsychological measure and the testing conditions should resemble requirements found in a patient’s activities of daily living (Spooner and Pachana, 2006). Early discussions of verisimilitude in neuropsychology emphasized that the technologies current to the time could not replicate the environment in which the behavior of interest would ultimately take place (Goldstein, 1996). Almost 20 years later, most neuropsychological assessments represent outdated technologies (e.g., paper and pencil assessments; static stimuli) that are yet to be validated with respect to real-world functioning (Rabin et al., 2007). Further, current verisimilitude assessments are somewhat conflicted in that while they focus on cognitive constructs (e.g., attention, executive function, memory), they are used for identifying functional abilities (Chaytor and Schmitter-Edgecombe, 2003).

##### Construct-driven measures without regard for prediction of “functional” behavior

In a more recent development of the ecological validity discussion Burgess et al. (2006) have argued that the majority of neuropsychological assessments currently in use today were developed to assess cognitive “constructs” (e.g., working memory) without regard for their ability to predict “functional” behavior. For example, one of the most widely used measures of executive function is the Wisconsin Card Sort Test (WCST; Heaton et al., 1993). The WCST (like many paper-and-pencil tests in use today) was not originally developed as a measure of executive functioning. Instead, the WCST was preceded by a number of sorting measures that were developed from observations of the effects of brain damage (e.g., Weigl, 1927). Nevertheless, in a single study by Milner (1963); patients with dorsolateral prefrontal lesions were found to have greater difficulty on the WCST than patients with orbitofrontal or nonfrontal lesions. However, the majority of neuroimaging studies have found activation across frontal and non-frontal brain regions and clinical studies have revealed that the WCST does not discriminate between frontal and non-frontal lesions (Stuss et al., 1983; Nyhus and Barceló, 2009). Further, while data from the WCST does appear to provide some information relevant to the constructs of set shifting and working memory, the data does not necessarily offer information that would allow a neuropsychologist to predict what situations in everyday life require the abilities that the WCST measures.

The WCST is not the only neuropsychological assessment to fall short of achieving ecological validity. Other tests such as the Stroop and Tower tests were also not developed to be used as clinical measures (Burgess et al., 2006). Instead, these measures were developed for cognitive assessments in nonclinical populations and only later found their way into the clinical realm. For example, neuropsychologists use the Stroop to assess the differences between performance on the automatic (e.g., word reading) and controlled (e.g., interference) conditions to make judgements about a patient’s difficulties with everyday functioning. The predictions are predominantly based on the assumption that the performance on a controlled process (inhibition of a prepotent response) reflects constructs that are important to carrying out real-world functions. A neuropsychologist may predict that a patient’s inability to inhibit an automatic response like reading words during the Stroop interference task means that the patient may also have problems inhibiting an impulse to put on the breaks if a traffic light turns red and the driver is already partway through an intersection (Marcotte and Grant, 2009).

##### Function-led tests that are representative of real-world functions

A more ecological approach to neuropsychological assessment is to move from construct driven assessments to tests that are representative of real-world functions and proffer results that are generalizable for prediction of the functional performance across a range of situations. According to Burgess et al. (2006); a function-led approach to creating neuropsychological assessments will include neuropsychological models that proceed from directly observable everyday behaviors backward to examine the ways in which a sequence of actions leads to a given behavior in normal functioning; and the ways in which that behavior might become disrupted. As such, he calls for a new generation of neuropsychological tests that are “function led” rather than purely “construct driven.” A number of investigators have argued that performance on traditional neuropsychological construct driven tests (e.g., Wisconsin Card Sorting Test, Stroop) has little correspondence to activities of daily living (Manchester et al., 2004; Sbordone, 2008; Bottari et al., 2009). According to Chan et al. (2008); most of these traditional construct driven measures assess at the veridicality level and do not capture the complexity of response required in the many multistep tasks found in everyday activities.

##### Real-world assessments using the multiple errands tasks: potential and limitations

A number of function-led tests have been developed that assess cognitive functioning in real-world settings. For example, Shallice and Burgess (1991) developed the Multiple Errands Test (MET) as a function-led assessment of multitasking in a hospital or community setting. Patients (e.g., patients with prefrontal injuries) perform a number of relatively simple but open-ended tasks (e.g., buying particular items, writing down specific information, traveling to a specific location) without breaking a series of arbitrary rules. The examiner observes the patient’s performance and writes down the number and type of errors (e.g., rule breaks, omissions). The MET has been shown to have increased sensitivity (over construct driven neuropsychological tests) to elicit and detect failures in attentional focus and task implementation. It has also been shown to be better at predicting behavioral difficulties in everyday life (Alderman et al., 2003). However, there are a number of unfortunate limitations for the traditional MET that are apparent in the obvious drawbacks to experiments conducted in real-life settings. Functional-led neuropsychological assessments can be time consuming, require transportation, involve consent from local businesses, costly, and difficult to replicate or standardize across settings (Rand et al., 2009; Logie et al., 2011). Further, there are times when function-led assessments in real-world settings are not feasible for patients with significant behavioral, psychiatric, or mobility difficulties (Knight et al., 2002).

In summary, early discussions of verisimilitude in neuropsychology emphasized that the technologies current to the time could not replicate the environment in which the behavior of interest would ultimately take place. Today, most neuropsychological assessments continue to represent outdated technologies and static stimuli that are yet to be validated with respect to real-world functioning. While much of the early discussion of ecological validity reflected an emphasis upon veridicality and verisimilitude, Burgess et al. (2006) have updated the discussion to include differentiating of construct driven assessments from function led neuropsychological assessments. While a number of function-led approaches have been developed, there are obvious drawbacks (time consuming, require transportation, involve consent from local businesses, costly, and difficult to replicate or standardize across settings) to experiments conducted in real-life settings.

#### Affective Neuroscience: Importance of Affective States for Cognitive Processing

Historically, much of psychology has been understood as comprising three related fields: cognition; affect; and motivation. Until recently, cognitive psychology has been the most extensively studied of the three, with affect and motivation being comparatively neglected (Baddeley, 1981). While cognition can be studied relatively easily in the laboratory, affect and motivation call for assessment of real world activities (Masmoudi et al., 2012). In recent years, this emphasis upon cognition alone has been challenged by a growing body of research into emotions (Stuss and Levine, 2002; Kohler et al., 2010). Cromwell and Panksepp (2011) describe the historically cognitive emphasis as a top-down (cortical → subcortical) perspective that could hinder progress in understanding neurological and psychiatric disorders. As an alternative, they emphasize inclusion of bottom-up (subcortical → cortical) affective and motivational state-control perspectives.

##### Dual process approach to affective neuroscience

According to Panksepp (2005); Panksepp (2007); progress in human neuroscience has resulted in an understanding that brain areas are involved in both non-salient “Cold” and affectively “Hot” cognitions. Panksepp has found support for this view in Goel and Dolan (2003) examination of the neural basis of emotionally neutral (“Cold”) and affectively salient (“Hot”) reasoning using event-related fMRI. Findings revealed that a reciprocal engagement of dorsolateral prefrontal cortex (dlPFC) for “Cold” and vmPFC for “Hot” processing provides evidence for a dynamic neural system for information processing. The pattern of which is robustly influenced by affective saliency. This approach emphasizes distinction between executive inhibition and reactive inhibition (Nigg, 2003). Executive inhibition involves top-down (cortical → subcortical) effortful neurcognitive processing aimed at inhibitory control (Nigg, 2000, 2003); and reactive disinhibition reflects bottom-up (subcortical → cortical) processes, in which the individual attempts to regulate behavior in affect-laden situations (Nigg, 2003, 2006). This affective neuroscience approach represents a more embodied view which accepts that cognitions are essentially related to both the participant’s neurology and the environments in which study participants operate (Panksepp, 2009, 2010).

Within affective neuroscience, the dual process approach of “Hot” and “Cold” processing is prevalent among human neuroscientists (Greene et al., 2001, 2004; Goel and Dolan, 2003; Kerr and Zelazo, 2004; Bechara and Damasio, 2005; Seguin et al., 2007; Chan et al., 2008; Brock et al., 2009; Potenza and de Wit, 2010; Rubia, 2011). With this prevalence in mind, this review focuses on studies that have explicitly measured and/or manipulated a factor commonly linked to “Cold” cognitive and/or “Hot” affective processing. The Cold cognitive control found in top-down executive functioning is contrasted with Hot affective aspects of cognitive control found in bottom-up processing (Zelazo et al., 2003). While Cold cognitive processing tends to be relatively logic-based and free from much affective arousal, Hot affective processing occurs with reward and punishment, self-regulation, and decision-making involving personal interpretation (Seguin et al., 2007; Chan et al., 2008; Brock et al., 2009). A number of studies have found that impairments in either the Cold or Hot cognitive functions may be related to deficits in everyday decision making and functioning (e.g., independance at home, ability to work, school attendance, and social relations; for review, see Chan et al., 2008).

##### Dual Process Approach, the Somatic Marker Hypothesis, and the Iowa Gambling Task

The idea of Hot decision making has been described as being consistent with the somatic marker hypothesis (Bechara and Damasio, 2005), in which the experience of an emotion (e.g., gut feeling; hunch) results in a somatic marker that guides choice of action. The somatic marker is hypothesized to play a role in Hot decision making in that it biases the available response selections found in decision making tasks. When persons are faced with decisions, they experience somatic sensations in advance of real consequences of possible different alternatives. The Iowa Gambling Task (IGT; Bechara, 2007) is a computerized assessment of reward-related decision-making that measures temporal foresight and risky decision-making (Bechara et al., 1994). Neuroimaging studies of persons performing the IGT have revealed activation in the orbitofrontal cortex (Grant et al., 1999; Ernst et al., 2002; Windmann et al., 2006), which appears to be significant for signaling the anticipated rewards/punishments of an action and for adaptive learning (Schoenbaum et al., 2011). Evidence for the somatic marker hypothesis’s role in Hot decision making over IGT trials can be found in the demonstration of an anticipatory electrodermal response in healthy controls to card selection (Bechara et al., 1996, 1997). For example, prior to selecting a card from a risky deck, a healthy control will show a physiological reaction indicating that the participant is experiencing bodily the anticipated risk. Further, studies have shown that damage to the ventromedial prefrontal cortex (vmPFC) and the amygdala prevents the use of somatic (affective) signals for advantageous decision making (Bechara et al., 1996, 1998; Bechara et al., 2000). It is noteworthy that there are different roles played by the vmPFC and amygdala in decision-making. While vmPFC patients were able to generate electrodermal responses when they received a reward or a punishment, amygdala patients failed to do so (Bechara et al., 1999). Whereas the IGT may have potential for assessment of “Hot” affective processing (Baddeley, 2012), researchers have argued that the IGT is deficient for understanding the affective impact of emotional stimuli upon cognitive processing because the observed effects on the IGT may simply be assessing cognitive (not affective) construct demands resulting from such a complex decision task (Hinson et al., 2002, 2003). Further, like other cognitive measures, the IGT was created to assess the construct of decision making in a laboratory setting, but it remains to be seen whether a relation between performance on the IGT and real-world decision making exists (Buelow and Suhr, 2009).

##### Dual process approach to moral decision making

Differentiating aspects of Cold cognitive processing and Hot affective processing are increasingly being found in moral decision making (Greene et al., 2001, 2004). Similar to neuroimaging studies into the somatic marker hypothesis using the IGT, the orbitofrontal cortex has been implicated in moral decision making. Lesions to the orbitofrontal cortex result in abnormal social behavior and deficits in moral decision making. (Berthoz et al., 2002; Takahashi et al., 2004). The affective neuroscience of ethical dilemmas is reflected in vignettes that challenge a participant to deliberate over intentionally harming an innocent character in order to promote the greater good of other characters in the vignette. This greater good reflects a utilitarian (i.e., consequentialist) decision to act in a way that will bring about the best overall consequences for a group of persons at the cost of a single individual’s wellbeing. Examples of moral decision making assessment include the Trolley and Footbridge Dilemmas, in which the participant reads a text in which a runaway trolley is heading for five immobile people on its tracks (Thomson, 1985; Greene et al., 2004). If the trolley is allowed to continue unmoved from its course, it will kill the five people. In the text of the Footbridge Dilemma, the participant is standing next to a very large stranger on a footbridge spanning the tracks through which the trolley will travel (Thomson, 1985; Greene et al., 2004). The participant’s only option for saving the five defenseless persons is to heave the large stranger off the footbridge. While this will kill the large persons, it also has the value of blocking the trolley from killing the five helpless persons. Participants reading these vignettes often find it difficult to decide that one answer (kill the large person to save the five others) is preferable to the other (saving the large person, but let the five die).

A dual-process theory has been proposed to describe the processes involved in resolving these moral dilemmas. According to the dual-process perspective both controlled cognitive responses and automatic affective responses perform essential roles in moral decision making (see also Greene et al., 2008): (1) Automatic and Hot affective processes drive non-utilitarian processes and reflect prohibition of harm, in which negative affective responses are generated in the medial prefrontal cortex and the amygdala (Greene et al., 2001, 2004) and (2) Controlled and Cold cognitive evaluations drive utilitarian judgments and weigh the costs and benefits associated with an action. Neuroimaging research has demonstrated that damage to the vmPFC results in moral decisions promoting harmful behavior when it will result in the promotion of a greater good (Ciaramelli et al., 2007; Koenigs et al., 2007). While judgments of correct actions when reading these hypothetical trolley dilemmas tend to involve controlled (e.g., Cold) cognitive processes, the decision to apply direct physical force triggers automatic (e.g., Hot) affective responses.

A limitation of these hypothetical moral dilemmas is that while they have been effective in enhancing our understanding of moral decision making, they do little to expand our knowledge of how these vignettes translate into real-world behaviors. While the use of text-based scenario descriptions (at times accompanied by a picture illustrating the scenario) and/or graphic-based questionnaires offer the experimental control found in laboratory-based assessment, the static presentation limits the contextual features of the dilemmas (Patil et al., 2014). Consequently, results from both paper-and-pencil and fMRI investigations may overestimate cognitive processes and underestimate important dynamic situational and affective components of the moral dilemmas (Bzdok et al., 2012).

#### Social Neuroscience and the Need for Dynamic Stimulus Presentations

Neisser (1982) call for cognitive assessments that can generalize beyond the narrow laboratory context has recently surfaced in the context of social neuroscience. Many of the pioneering paradigms in social neuroscience reflect a noteworthy emphasis upon laboratory control and experiments that involve participants observing static stimuli (e.g., simple, static representations of socially relevant stimuli; static photographs of emotionally valenced facial expressions) that are devoid of interactions (Morris et al., 1996; Blair et al., 1999). Although social neuroscience researchers have assumed that knowledge gained using these static stimuli will generalize to the social cognition in everyday activities, a number of researchers are beginning to question this assumption (Ochsner, 2004; Schilbach et al., 2006, 2013; Chakrabarti, 2013). This has led to confusion about the neural bases of interpersonal understanding because studies of shared representations between self and others usually ask the perceiver to observe and imitate target movements or have participants directly rate their own sensory states and observe those states in others. This is done without requiring participants to make any judgments about target states. Further, researchers examining the shared representations and mental state attributions of perceivers often make use of static stimuli that fail to represent the types of social information that participants must process when they experience dynamic stimuli in real-life social interactions (Zaki and Ochsner, 2009; Risko et al., 2012; Schilbach, 2015).

##### Differences between real-life social information and laboratory stimuli

Zaki and Ochsner (2009) stress three critical ways that real-life social information differ from laboratory stimuli. Specifically, they discuss the ways in which cues about target states in the real world are: (1) Multimodal (include visual, semantic, and prosodic information); (2) Dynamic in that stimuli are presented serially or concurrently to participants over time; and (3) Contextually embedded so that participants are presented with stimuli and environmental information that can frame their interpretation of another’s internal states. Multimodal stimuli are important for an ecologically valid social neuroscience because social mentalizing and interactions occur in situations that involve the convergence of multiple channels such as auditory perception of social cues (verbal utterances, intonation, prosody); visual perception of social cues (nonverbal communication, gestures, postures, facial expressions); and emotional perception (positive and negatively valenced representations of the other’s internal states). Zaki and Ochsner (2009) emphasize the importance of the emotional modality because early neuroimaging studies of emotion treated cognitive phenomena as qualities (shape, size, or color) of a stimulus. They give the example of showing participants negatively valenced stimuli (e.g., gruesome picture) and then infer that this caused the participant to experience negative affect without actually measuring the participant’s subjective experience or other behavioral indices of emotional responding.

##### Need for dynamic stimuli

There is also need for greater emphasis upon dynamic stimuli that will allow assessment of participants as they act upon stimuli interactively instead of a passive response to static stimuli. While limiting stimuli to static representations with constrained variance along tractable dimensions is important for maintaining experimental control over the social cognitive processes studied in an experiment, such experimental constrictions can result in artificially constrained understandings of the social cognitive processes involved. There is a need to emphasize dynamic stimuli that reflect real-life interactions in which participants react to dynamic stimuli in an interactive way that modifies subsequent dynamic stimuli. An unfortunate limitation of many social neuroscience approaches is that the participant is presented static and controlled stimuli that lead the participant to believe and act “as if” the participant could modify the course of a social interaction. As such, many current social neuroscience approaches do not include real-time dynamic and adaptive virtual agents with complex cognitive architectures (Faur et al., 2013). Although advances in technologies now allow dynamic interactions with intelligent virtual agents, many constraints on experimental designs and subsequent statistical analyses often restrict the stimuli to more traditional approaches.

##### Contextualized succession of events

In addition to multimodal and dynamic stimuli, ecologically valid social neuroscience paradigms also involve the contextualized succession of events that are intuitively clear when associated within a sequence. While many social neuroscience approaches use various stimulus approaches (comic-strips, video-based mental state inferences) to depict an agent performing actions following a specific schema, they often do not promote a context that will prompt the participant to have an invested interest in this agent. Without adequate social context, the participant may not enter experience an empathetic relationship with the agent. Further, given the assumption that social interaction may be experienced as motivating and rewarding, social neuroscience approaches should implement a social context that allows for assessment of motivations to interact socially. Social neuroscience experiments place participants in a scanner and instruct them to fixate on stimuli presented on a monitor. Neuroimaging resulting from such studies appears to be significantly dissimilar to studies in which the participant is performing such tasks together with another agent. The addition of another agent results in a differential increase of neural activity in brain areas that have been related to grasping another’s mental states (Schilbach et al., 2008).

##### Social interactions found in everyday exchanges

The social interactions found in everyday exchanges involve a broad array of contexts, including verbal and nonverbal interactions, interpretation of others, representations of self and other, and joint attention. In order to understand social interactions, social neurosciences should have the methods necessary for assessing self-understanding, self and other interactions, and relations between self and other in everyday environments (Sebanz et al., 2006; Schilbach et al., 2013). An unfortunate limitation for many social neuroscience approaches is that they study a single participant’s brain in isolation instead of during real-world interactions (Tanabek et al., 2012). For example, participants are often isolated from their natural environments and placed into a sealed room where the participant is exposed to static images, auditory stimuli, and/or video clips. Social neuroscientists have designed a number of innovative paradigms to investigate the neural bases of various aspects of social interactions: (1) the participant views video clips and/or pre-recorded interactions (Iacoboni et al., 2004; Walter et al., 2004); (2) the participant takes part in an online game (e.g., Cyberball) with an avatar that is playing catch with two other avatars (Lieberman and Eisenberger, 2009; Eisenberger, 2012); or (3) the participant views a noninteractive virtual human that shifts gaze towards or away from the participant (Pelphrey et al., 2003, 2004). While these methodological approaches afford significant information related to the potential neural underpinnings of social interaction, they are missing essential mechanisms of everyday social interactions (Hari and Kujala, 2009). The difficulties in either developing laboratory controlled experiments or naturalistic interactions between two participants are apparent in fMRI studies (Montague et al., 2002).

In summary, the social interactions found in everyday exchanges involve a broad array of contexts, including verbal and nonverbal interactions, interpretation of others, representations of self and other, and shared attention. In order to understand social interactions, it is important that social neurosciences have the methods necessary for assessing self-understanding, self and other interactions, and relations between self and other in everyday environments (Sebanz et al., 2006; Schilbach et al., 2013). An unfortunate limitation of social neuroscience assessments is that it is difficult to generate study findings that can generalize beyond the narrow laboratory context. Many of the pioneering paradigms in social neuroscience involve observing static stimuli (e.g., simple, static representations of socially relevant stimuli; static photographs of emotionally valenced facial expressions) that are devoid of interactions. While there are a number of innovative paradigms to investigate the neural bases of various aspects of social interactions, they are missing essential mechanisms of everyday social interactions.

### Virtual Reality for Enhanced Ecological Validity and Experimental Control

Given that virtual environments represent a special case of computerized neuropsychological assessment devices (Bauer et al., 2012) they have enhanced computational capacities for administration efficiency, stimulus presentation, automated logging of responses, and data analytic processing. This greater computation power results in enhanced capacity for generating perceptual environments that systematically present and record neurobehavioral responses to dynamic stimuli. Advances in virtual environment technology offer platforms in which three-dimensional objects are presented in a dynamic, consistent, and precise manner. A virtual environment provides the researcher with an ecologically valid platform for presenting dynamic stimuli in a manner that allows for both the veridical control of laboratory measures and the verisimilitude of naturalistic observation of real life situations (Matheis et al., 2007; Jovanovski et al., 2012a, b). Virtual environment-based assessments can provide a balance between naturalistic observation and the need for exacting control over key variables (Campbell et al., 2009).

Virtual environments allow researchers to immerse participants in simulations that produce a sense or presence (e.g., “being there”). The ideas of presence and immersion have been explicated by Rebelo et al. (2012) as key concepts to understanding the psychological and physical experiences of participants using. They describe immersion as being characterized by the participant’s perception of being inserted into an environment, while being directly connected to the virtual surroundings and the sensations (e.g., visual, auditory, haptic) and feelings that result. As such, “presence” is connected to the psychological features involved and is confirmed in the sensations (visual, auditory, haptic) activated in the participant’s relationship with the virtual environment.

While there are a number of advantages inherent in virtual environments, there are some drawbacks that researchers should consider when they are contemplating the adoption of virtual environments for their studies: cost, bulkiness of equipment, simulator sickness, specialist technology skills, and psychometric validation. Cost has long been a barrier to incorporating advanced virtual environments. While a wide-field-of-view head-mounted display with tracking capabilities can cost thousands of dollars, computer equipment setups are rapidly decreasing in size and price—with a concomitant increase in computational power and ease of operation. Further, these systems have historically been bulky HMD helmets that engulfed the user’s entire head and face, and weighed several pounds. Given progress in the design of HMDs, these problems have steadily diminished. For example, the Oculus Rift (Oculus, USA) is a new and light weight (380g) head mounted display that only costs a few hundred dollars (i.e., $300) and it proffers an extended field of view of 110 degrees, stereoscopic vision, and rapid head tracking. The Oculus Rift has advanced data processing of stimuli that comes through 3-axis gyroscope, accelerometer, and magnetometer, giving the user a fast image update with greatly limited delay. Nevertheless, simulator sickness continues to be a potential issue for some users. Simulator sickness is typically understood as nausea resulting from the incongruity between visual perception of motion and vestibular feedback that is not matched by vision (Kennedy et al., 2000; Keshavarz and Hecht, 2011). Another issue that is commonly raised is the need for technology skills for creation and maintaining virtual environments. This issue is becoming less formidable as increasingly powerful tools are becoming available. In addition to the fact that many of these tools are free to users (e.g., Unreal, Unity, Source 2), large repositories of graphic assets are increasingly available for use in user friendly visual programming and scripting languages (e.g., Virtools and Vizard). A final concern for researchers wanting to incorporate virtual environments is that of psychometric validity. While a number of studies have attempted to establish the validity of these environments via comparisons with well-established paper-and-pencil as well as computer automated platforms, results reveal the need for judicious use of these virtual platforms. Researcher must carefully judge the degree to which the virtual environments offer something beyond less expensive and modalities that are free from technological and simulator sickness issues.

In summary, virtual environment-based assessments allow for real-time assessment of a participant’s clinical, affective, and social processing in a manner that more closely resembles real-world functional abilities (Parsey and Schmitter-Edgecombe, 2013). While many of the limitations inherent in virtual environment technology have been addressed via advances in technology, researchers need to consider the potential remaining limitations (e.g., simulator sickness for some cohorts and validation) before incorporating them into an experimental paradigm. In the following, there will be a description of the potential of virtual reality for precise presentation and control of dynamic perceptual stimuli that can be used for assessment of neurocognitive, affective, and social processing while participants are immersed in simulations of real world contexts. Further, there will be a discussion of both strengths and potential limitations of some virtual environments.

#### Virtual environments for clinical neuroscience

A difficulty for most neuropsychological assessments is that performance on tests (e.g., WCST; Stroop task) of a given construct (e.g., working memory) may have little or no predictive value for how a person functions in a real world situation (Chaytor et al., 2006). Neuropsychologists are more and more emphasizing the need for tasks that represent real world functioning and tap into a number of cognitive domains (Chaytor and Schmitter-Edgecombe, 2003). Virtual environments are increasingly considered as potential aids in enhancing the ecological validity of neuropsychological assessments (Campbell et al., 2009; Renison et al., 2012). While early virtual reality equipment suffered a number of limitations (large and unwieldy, difficulty to operate, and expensive to develop and maintain), today’s virtual environment systems are more reliable, cost effective, and acceptable in terms of size and appearance (Bohil et al., 2011). In this section, both construct-driven and function-led virtual environments will be discussed. First, given this review’s emphasis upon the need for function-led assessments for ecological validity, there will be a discussion of early virtual environments that focused on automating paper-and-pencil version of construct driven tests (e.g., WCST). While these early environments were novel in their approach, they added little to paper-and-pencil assessments and suffered the limits of the paper-and-pencil analogs. Next, newer construct-driven virtual environments are presented that have the advantage of distractors for increasing ecological validity. Unfortunately, the addition of distractors does little to increase ecological validity or move beyond paper-and-pencil approaches. Finally, function-led virtual environments will be described that greatly enhance ecological validity via an emphasis proceeding from directly observable everyday behaviors and moving backward to examine the ways in which a sequence of actions leads to a given behavior in normal functioning.

##### Virtual environments with construct driven veridicality assessments

Early virtual environments attempted to build upon the construct-driven neuropsychological assessments found in traditional paper-and-pencil assessments. For example, a number of early virtual environments aimed to build upon the WCST paradigm, in which participants navigated to the exit of a virtual building through the matching of stimuli to environmental cues (e.g., categories of shape, color, and number of portholes) found on virtual doors (Pugnetti et al., 1995, 1998). A notable limitation of these early virtual environment was a heavy reliance on navigating through a building and this may have confounded the results. In a novel and evolved iteration, the Virtual Reality Look for a Match (VRLFAM) test removed the navigational components and immersed the participant in a virtual beach scene. Participants were instructed to deliver frisbees, sodas, popsicles, and beach balls to umbrellas (Elkind et al., 2001). While these virtual environments were innovative approaches, an unfortunate limitation of modeling virtual environment-based neuropsychological assessments off of the WCST is that the virtual analogs, like the original WCST, may not be able to differentiate between patients with frontal lobe pathology and control subjects (Stuss et al., 1983).

##### Virtual environment-based distractors to enhance construct driven tests

A more recent approach to developing virtual environments has involved the addition of ecologically valid distractors (e.g., phone ringing, car driving by) to construct-driven stimuli (e.g., Stroop stimuli) presented in the virtual environment. For example, the Virtual Apartment presents Stroop stimuli on a large television set in the living room while the phone is ringing (Henry et al., 2012). The addition of distractors into the virtual environment allows traditional cognitive constructs to be assessed with external interference and the distracters (auditory and/or visual elements). Virtual environment-based distracters pull for head movements that allow a better detection of subtle deficits (Henry et al., 2012). As a result, a main advantage of these virtual environments is that they allow the researcher to introduce distractors into a task that is more similar to the real world functioning. A number of virtual classroom environments have emerged that include distractors (Climent and Bánterla, 2011; Iriarte et al., 2012; Lalonde et al., 2013; Díaz-Orueta et al., 2014). In these virtual classrooms the participant is seated at one of the desks and is surrounded by desks, children, a teacher, and a white board much like they would be in a real-world classroom. Various construct driven tasks can be presented on the whiteboard in the front of the room and the participant performs a task (e.g., Stroop or continuous performance tasks, CPTs) with auditory (e.g., airplane passing overhead, a voice from the intercom, the bell ringing) and visual (e.g., children passing notes, a child raising his hand, the teacher answering the classroom door, principal entering the room) distractors in the background.

In a clinical trial of a Virtual Classroom with an embedded CPT, Parsons et al. (2007) compared performance of children with attention deficit/hyperactivity disorder (ADHD; *N* = 9) with typically developing children (*N* = 10). In this study, children with ADHD performed differently from typically developing children in a number of different ways: (1) children with ADHD made more commission and omission errors; (2) children with ADHD exhibited more overall body movement; and (3) children with ADHD were more impacted by distracting stimuli. Comparison of children with ADHD and matched controls using the Virtual Classroom CPT and the traditional CPT have revealed consistent findings (Adams et al., 2009; Pollak et al., 2010; Bioulac et al., 2012). A limitation of these studies is that the Virtual Classroom CPT simply replicated effects found in the traditional Conner’s CPT. As a result, the Virtual Classroom did not add to our understanding attentional processing. That said, the Parsons et al. (2007) study using the Virtual Classroom paradigm allowed for tracking of body movement and revealed that the addition of distractors increased body movement significantly more in the children with ADHD. The ability of the Virtual Classroom to quantify body movement represents a potential advance over subjective rating scales for identification of hyperactivity. Nevertheless, there is need for virtual environment measures that do more than correlate with traditional construct-driven measures.

##### Virtual environments designed for use as function-led assessments

In the clinical neurosciences there is increasing interest in neuropsychological assessments that are representative of real-world functions. These function-led assessments are developed by proceeding from directly observable everyday behaviors backward to examine the ways in which a sequence of actions leads to a given behavior in normal functioning. While a number of function-led assessments in naturalistic environments have been developed, they face a number of limitations: time consuming, require transportation to the location, involve consent from local businesses, costly, and difficult to replicate or standardize across settings (Rand et al., 2009; Logie et al., 2011). Further, there are times when function-led assessments in real-world settings are not feasible for participants with significant behavioral, psychiatric, or mobility difficulties (Knight et al., 2002). To overcome some of these concerns, McGeorge et al. (2001) modeled a Virtual Errands Test (VET) off of the original MET and found performance was similar for real-world and virtual environment tasks. In a larger study, the VET scenario successfully differentiated between participants with brain injuries and normal controls (Morris et al., 2002). An unfortunate limitation of these early virtual environments is that they included unrealistic graphics without event-based logging. Instead, assessment of performance involved the video recording of test sessions with subsequent manual scoring.

###### Virtual environments that reflect real-world tasks

A number of newer virtual environments with realistic graphics and event-based logging have been modeled off of the MET. These function-led virtual environments have been created and validated in clinical samples. Given that these virtual environment protocols were developed by proceeding from directly observable everyday behaviors backward they examine the ways in which a sequence of actions leads to a given behavior in living and work settings: Virtual Office (Lamberts et al., 2009; Montgomery et al., 2011; Jansari et al., 2013); Virtual Apartment/Home tasks (Sweeney et al., 2010); Virtual Park (Buxbaum et al., 2012); Virtual Library Task (VLT; Renison et al., 2012); Virtual Anticipating Consequences Task (Cook et al., 2013); Virtual Street Crossing (Clancy et al., 2010); and Virtual Kitchen (Cao et al., 2010). Further, there are a number of virtual environment-based neuropsychological assessments that use driving simulators (Schultheis et al., 2007; Asimakopulos et al., 2012; Calhoun and Pearlson, 2012).

While there are a number of virtual environments available for function-led assessment, the Multitasking in the City Test (MCT) is especially useful because it involves an errand-running task implemented in a virtual city (Jovanovski et al., 2012a, b). The MCT is made up of a virtual city that includes a post office, drug store, stationary store, coffee shop, grocery store, optometrist’s office, doctor’s office, restaurant/pub, bank, dry cleaners, pet store, and the participant’s home. While immersed in the MCT, participants are assessed on their planning ability, self-monitoring, multitasking, prioritization of competing subtasks, and utilization of feedback to guide decision making. The MCT errands consisted of everyday tasks such as meeting deadlines, making purchases, and staying within a budget. While the MCT is modelled off the real-world MET, it differs in that the MCT intentionally was developed with as a less structured task without the explicit rules that may constrain behavior in the MET. Instead, the MCT tasks were developed to more closely resemble everyday behaviors. Further, unlike most versions of the MET that have no opportunity to plan tasks ahead of time, planning in the MET is assessed and compared to actual task performance. In a study with 30 healthy participants, Jovanovski et al. (2012a) found that the MCT may provide an ecologically valid method of objectively evaluating the integration of component executive functions into meaningful behavior. This represents a departure from traditional paper-and-pencil measures that aim to assesses cognitive constructs (e.g., working memory) without reference to real-world settings. Further, using the MCT researchers were able to compare a sample of post-stroke and traumatic brain injury (TBI) patients to an earlier sample of normal controls. Jovanovski et al. (2012b) found that while participants in the clinical sample were able to develop adequate strategies for task execution, the actual completion of the tasks revealed a greater number of errors. The MCT’s capacity for assessing subprocesses not tapped into by traditional measures is apparent in that most participants made errors during the MCT despite having successfully completing all tasks. For example, participants often attempted to make a purchase before obtaining money from the bank, they entered a building but failed to perform the required task within the building, entered the same building on two separate occasions to perform two tasks that could have been performed in a single visit, and entered unnecessary buildings. Participants were also evaluated on their devising of which route they would take to complete errands around the city prior to task initiation. These findings move beyond paper-and-pencil measures and highlight the importance of prior planning for efficient and successful goal-directed behavior.

###### Comparison of real-world assessments to virtual-world assessments

A further example of function-led virtual environments is the VLT. During the VLT, participants are required to perform numerous indicated tasks associated with the day to day running of the library, while observing predetermined rules: cool the library navigating to the air conditioner adjusting the controls; problem solve an alternative method to cool the room if the air conditioner is broken, check items that appear in the in-box; and move objects to desired locations. Renison et al. (2012) aimed to investigate whether performance on a VLT was similar to performance of the same task in a real-world library. Findings revealed that scores on the VLT and the real world library task were highly positively correlated, suggesting that performance on the VLT is similar to performance on the real world library task. This finding is important because the virtual reality environment allows for automated logging of participant behaviors and it has greater clinical utility than assessment in real world settings. Comparisons of persons with TBI and normal controls supported the construct validity of the VLT as a measure of executive functioning. In fact, the VLT was found to be superior to traditional (e.g., WCST) tasks in differentiating between participants with TBI and healthy controls. For example, the WCST failed to significantly differentiate between the two groups. This is consistent with studies that have reported no significant differences between control and brain injured performances on the WCST (Alderman et al., 2003; Dawson et al., 2009; Ord et al., 2010). The authors contend that the disparity between the demands of functional assessments and traditional testing environments most likely accounts for the differences (Manchester et al., 2004).

In summary, a number of researchers have emphasized convergent validity between construct-driven virtual environments and paper-and-pencil assessments. That said, little is gained from the use of virtual environments when a paper-and-pencil measure can already answer the neuropsychologists questions. The inclusion of distractors in construct driven virtual environments may enhance the ability to quantify body movement and perhaps diagnosis of “hyperactivity” in ADHD. However, this addition may not be enough to compel neuropsychologists to adopt new technologies. Greater enthusiasm is apparent for function led virtual environments that were developed by proceeding from directly observable everyday behaviors backward to examine the ways in which a sequence of actions leads to a given behavior in normal functioning. While these virtual reality-based function led assessments are at times correlated with traditional neuropsychological assessments, the most promising results have come from the increased understandings of the ways in which persons interact in everyday activities. They represent ecologically valid tasks that assess cognition and include the demands that participants face in the real world. It is important to note that the function led assessments need not replace tradition neuropsychological batteries. Instead, function-led virtual environment-based tests may be seen as an addition to current batteries. These virtual environments may allow for increased knowledge of competence in everyday functioning.

#### Virtual Environments for Affective Assessments

In addition to the cognitive processing assessments mentioned above, virtual environments are also being used to assess affective processes. Virtual reality has recently become an increasingly popular medium for assessment of various aspects of affective arousal and emotional dysregulation (Parsons and Rizzo, 2008). Virtual environments can be used when real-world exposure would be too costly, time consuming, or hazardous. It has been found to be an especially useful modality for assessing psychophysiological and behavioral responses of participants (Fahrenberg et al., 2007; Betella et al., 2014). In the following, a number of application areas for affective arousal using virtual environments are discussed: general fear conditioning; affective responses in everyday contexts; affective responses in threatening contexts; and affective processing of moral dilemmas. These areas were chosen because they cover the range of contexts for affective responding from simple fear conditioning to real world moral dilemmas.

##### Virtual environments for studies of fear conditioning

One application of interest for affective neuroscience is the use of virtual environments for studies of fear conditioning (Alvarez et al., 2007; Baas et al., 2008). Virtual environments offer an ecologically valid platform for examinations of context-dependent fear reactions in simulations of real-life activities (Mühlberger et al., 2007; Glotzbach et al., 2012). Neuroimaging studies utilizing virtual environments have been used to delineate brain circuits involved in sustained anxiety to unpredictable stressors in humans. In a study of contextual fear conditioning Alvarez et al. (2008) used a Virtual Office and fMRI to investigate whether the same brain mechanisms that underlie contextual fear conditioning in animals are also found in humans. Results suggested that contextual fear conditioning in humans were consistent with preclinical findings in rodents. Specifically, findings support Hot affective processing in that the medial aspect of the amygdala had afferent and efferent connections that included input from the orbitofrontal cortex. In another study using a Virtual Office, Glotzbach-Schoon et al. (2013) assessed the modulation of contextual fear conditioning and extinction by 5HTTLPR (serotonin-transporter-linked polymorphic region) and NPSR1 (neuropeptide S receptor 1) polymorphisms. Results revealed that both the 5HTTLPR and the NPSR1 polymorphisms were related to Hot affective (implicit) processing via a fear potentiated startle. There was no effect of the 5HTTLPR polymorphism on Cold cognitive (explicit) ratings of anxiety. Given the ability of virtual environments to place participants in experimentally controlled yet contextually relevant situations, there appears to be promise in applying this platform to future translational studies into contextual fear conditioning.

##### Virtual environments to elicit affective responses in everyday contexts

The use of virtual environments has also been used to elicit affective responses in everyday contexts. For example, Riva et al. (2007) manipulated virtual parks to elicit affective arousal. All participants experienced anxious, relaxing and neutral parks. Although all the same structure was found in all three parks, the parks differed in the aural and visual experience that was presented to the participant. Findings suggested that the three affective park presentations (anxious and relaxing) have some efficacy for eliciting specific emotional states. In related studies virtual environments have successfully elicited heightened affective arousal in a virtual train (Freeman et al., 2008) and a virtual office (Roy et al., 2003; Klinger et al., 2005). These results suggest that virtual environments can be used as an affect induction medium. In another virtual office scenario participants were immersed in a virtual office scenario designed to elicit anger (Macedonio et al., 2007). Within a few seconds of starting the virtual environment, a virtual boss approaches and verbally confronts the participant in a hostile and condescending fashion. The study found physiological correlates of anger arousal stimuli from a virtual environment. In a more fully developed study Mühlberger et al. (2008) matched arousal events in a Virtual Tunnel driving experiment with startle reflex methodology to investigate whether the phylogenetically relevant aversive context of darkness elicited fear responses. Results revealed increased negative affect during darker areas of the virtual tunnel. The increased logging abilities of these virtual environments allow for matching psychophysiological responses to events as they occur in the simulations.

##### Virtual environments to elicit affective responses in threatening contexts

Recently, virtual environments have been applied to the assessment of both “Cold” and “Hot” processes using combat related scenarios (Armstrong et al., 2013; Parsons et al., 2013). The addition of virtual environments allows affective neuroscience researchers to move beyond the ethical concerns related to placing participants into real-world situations with hazardous contexts. The goal of these platforms is to assess the impact of Hot affective arousal upon Cold cognitive processes. For example, Parsons et al. (2013) have developed a Virtual Reality Stroop Task (VRST) in which the participant is immersed in a simulated High Mobility Multipurpose Wheeled Vehicle (HMMWV) and passes through zones with alternating low threat (driving down a deserted desert road) and high threat (gunfire, explosions, and shouting amongst other stressors) while dual-task stimuli (e.g., Stroop stimuli) were presented on the windshield. They found that the high-threat zones created a greater level of psychophysiological arousal (heart rate, skin conductance, respiration) than did low threat zones. Findings from these studies also provided data regarding the potential of military relevant virtual environments for measurement of supervisory attentional processing (Parsons et al., 2013). Analyses of the effect of threat level on the color–word and interference scores resulted in a main effect of threat level and condition. Findings from the virtual environment paradigm support the perspective that: (1) high information load tasks used for Cold cognitive processing may be relatively automatic in controlled circumstances—for example, in low threat zones with little activity; and (2) the total available processing capacities may be decreased by other Hot affective factors such as arousal (e.g., threat zones with a great deal of activity). In a replication study, Armstrong et al. (2013) established the preliminary convergent and discriminant validity of the VRST with an active duty military sample.

In addition to virtual environment-based neuropsychological assessments using driving simulators, a number of other military relevant virtual environments have emerged for neurocogntive assessment of Cold and Hot processes. For example, (Parsons et al., 2012; Parsons and Courtney, 2014) immersed participants into a Middle Eastern city and exposed participants to a Cold cognitive processing task (e.g., paced auditory serial addition test) as they followed a fire team on foot through safe and ambush (e.g., Hot affective—bombs, gunfire, screams and other visual and auditory forms of threat) zones in a Middle Eastern city. In one measure of the battery, a route-learning task, each zone is preceded by a zone marker, which serves as a landmark to assist in remembering the route. The route-learning task is followed immediately by the navigation task in which the participants were asked to return to the starting point of their tour through the city. Courtney et al. (2013) found that the inclusion of Hot affective stimuli (e.g., high-threat zones) resulted in a greater level of psychophysiological arousal (heart rate, skin conductance, respiration) and decreased performance on Cold cognitive processes than did low threat zones. Results from active duty military (Parsons et al., 2012) and civilian (Parsons and Courtney, 2014) populations offer preliminary support for the construct validity of the VR-PASAT as a measure of attentional processing. Further, results suggest that the VR-PASAT may provide some unique information related to Hot affective processing not tapped by traditional Cold attentional processing tasks.

##### Virtual reality-based moral dilemmas

Virtual environments are also being applied to the affective neuroscience of moral decision making. Recently studies have emerged that take the classic Trolley Dilemma and modify the text-based approach via a Virtual Trolley Dilemma (Pan and Slater, 2011; Navarrete et al., 2012; Patil et al., 2014; Skulmowski et al., 2014). Further, while text-based hypothetical moral dilemmas led to gaps in our understanding of how results translate into real-world behaviors, virtual environments allow for observations of morally relevant decision making behaviors in realistic three-dimensional simulations. With virtual environments, researchers can perform real-time assessment of the cognitive and affective factors inherent in explicit moral behaviors. Patil et al. (2014) compared traditional text-based approaches to a virtual environment version of the trolley dilemma. They found a modality specific difference in that participant behavior in the virtual environment reflected a utilitarian approach, but in the text-based descriptions the same moral dilemmas resulted in nonutilitarian decisions. Further, autonomic arousal was greater in virtual environments. These differences suggest that text-based scenario presentation does not include dynamic visual information that is available to persons in real-world environments. With virtual environments there appears to be enhanced capacity for the context-dependent knowledge that is critical for moral decision making.

Navarrete et al. (2012) used virtual environments to observe behaviors and record autonomic arousal of participants as they confronted moral dilemmas. Specifically, they immersed participants into a virtual reality version of the trolley problem. Participants were given the choice of whether or not to pull a lever that would determine the fate (e.g., death or safety) of some number of people. The virtual environment included virtual human agents that were capable of movement and sound in real time. Validity of the virtual trolley paradigm was apparent in that results were consistent with the behavioral pattern observed in studies using text versions of the trolley dilemma. Results also revealed that affective arousal was: (1) associated with a reduced likelihood that participants were acting to achieve a utilitarian outcome; and (2) greater when participants were attempting to behaviorally resolve a dilemma that required committing an act than when participants were omitting an action. An important aspect of these findings is that they provide support for a relation between Hot affective processing and moral action. These findings also suggest that similar neurophysiological processes may mediate Cold processing of moral judgments and actions. Virtual environment based moral dilemmas appear to offer an empirical platform for investigating the contents and contexts in which Hot affective and Cold cognitive processing occur.

In a study that builds on Navarrete et al.’s (2012) paradigm, Skulmowski et al. (2014) developed a virtual reality-based trolley dilemma that utilized a first person perspective of the forced-choice decision-making paradigm. A novel aspect of the Skulmowski design is that the participants were the drivers of the train. This approach was chosen because research on presence and immersion in virtual environments has found that first-person perspectives elicit a greater sense of presence and involvement (Kallinen et al., 2007; Slater et al., 2010). The study also included psychophysiological assessment metrics drawn from pupillometry that were integrated into the virtual environment paradigm. Like Navarrete et al.’s virtual trolley study, Skulmowski’s experiment replicated the behavioral pattern found in studies using text-based versions of the trolley dilemma. This further validated the use of the virtual trolley platform for research on moral decision making. Additional findings included a peak in the level of arousal related to the moment that the moral decision was made. Further, eye-tracking revealed context-dependent gaze durations during decisions to sacrifice. These findings comport well with dual-process theories. Since decision time frames were able to be held constant in the virtual environment paradigm, events could be logged and marked for comparison to pupillometric measurements. This approach offers promise for moving beyond paper-and-pencil (e.g., text-based) approaches in which participants read scenario descriptions at varying speeds.

In summary, mere judgments about moral dilemmas results in a limited understanding. The hypothetical and text based vignettes attempt to stimulate the imagination of participants and then use questionnaires or experiments involving low-level manipulations of harm to enhance understanding. The addition of virtual environments allows researchers to assess the expression of decision making processes via real time logging of behaviors. Given that virtual environments are more dynamic than text based scenarios and that they do not involve the potential for harmful outcomes, they may bridge the gap between judgment and behavior via explorations of the underlying mechanisms. While the virtual environment approach does not offer a definitive solution to the long-standing trade-off between laboratory control and real world behaviors, it does allow researchers a methodology for presenting participants with auditory and visual representations of real-world activities.

#### Virtual Environments for Social Neurosciences

In recent years there has been an increased interest in the application of neuropsychological and neuroscientific methodologies to research in contemporary social psychology (Lieberman et al., 2007; Adolphs, 2009, 2010). Although cognitive neuroscience has made gains in the exploration of the brain systems engaged when an individual shares and makes inferences about the internal states of others, they have emphasized isolation paradigms, that rely on participant observations of others behaviors and to make inferences about the mental states of others (Becchio et al., 2010). Recently, there has been a call to move from the third person mentalizing approach to a second person neuroscience that involves social interaction (Schilbach et al., 2013). Furthermore, researchers from the cognitive neurosciences typically use divergent and highly simplified stimuli and methods. As a result, research drawn from cognitive neuroscience studies using a strictly laboratory focused approach have produced largely non-overlapping results and artificially constrained social theories (Zaki and Ochsner, 2009). While many of the pioneering paradigms in social neuroscience have used static stimuli to study social cognition in everyday activities, a number of researchers are beginning to question this approach (Schilbach et al., 2006; Risko et al., 2012; Chakrabarti, 2013). While video recordings, movies, and imagery techniques have been used by social neuroscientists to elicit emotions (Zaki and Ochsner, 2009), enhanced ecological approaches increase the capacity to manipulate the content of interactive media to induce specific emotional responses. As Neisser (1980) argued, participants observing video-recordings of others, and then making judgments of what they saw miss an important interactive component that occurs in a social exchange. Virtual environments offer the social neuroscientist the ability to induce a feeling of presence in participants as they experience emotionally engaging background narratives to enhance affective experience and social interactions (Gorini et al., 2011; Diemer et al., 2015). Recently, social neuroscientists have started incorporating virtual reality into their experiments and are increasingly using virtual-reality stimuli in social neuroscience research (Adolphs, 2003; Wilms et al., 2010; Schilbach et al., 2013). In addition to advanced presentation of dynamic stimuli, virtual environments allow for moment by moment logging of interactive scenarios that comport well with the constraints of neuroimaging settings. As such, virtual environments offer promise for advancing the investigation of the neural underpinnings of joint actions (Schilbach et al., 2006, 2010; Newman-Norlund et al., 2008; Kokal et al., 2009; Pfeiffer et al., 2013). With advances in simulation technologies, the trade-off between the experimental control found in the laboratory and the ecological validity of naturalistic observation may be alleviated, as virtual technology can be modified and adapted without compromising measurement control (Bohil et al., 2011).

##### Neural underpinnings involved in interpreting others

A number of studies have emerged using virtual reality, psychophysiology, and neuroimaging of brain activity during naturalistic, face-to-face social interactions. These studies have aided the identification of brain regions involved in interpreting others’ face and eye movements. When participants were approached by a virtual human exhibiting an angry expression, researchers found activation of the superior temporal sulcus, the lateral fusiform gyrus, and a region of the middle temporal gyrus (Carter and Pelphrey, 2008). In other eye-gaze paradigms, (Schilbach et al., 2006, 2010) characterized the neural correlates of being involved in social interactions through the introduction of dynamic virtual humans in the scanner. The virtual humans were programmed to “gaze at” and “greet others.” The “others” were lying passively in a scanner or a bystander. Findings revealed that the vmPFC underpins the perception of social communication and feeling of personal involvement (Schilbach et al., 2006). Further, when the participants initiated shared attention with the virtual humans, neural activity increased in the ventral striatum (Schilbach et al., 2010). In a related study, Wilms et al. (2010) instructed participants in the scanner to “respond to” or “probe” the gaze of a virtual human that was being operated by another human. They were not told that the virtual human was in fact being operated by a computer. The goal of the participants was to establish eye contact with the virtual human and to attend jointly to one of three objects on a screen. The participants were instructed that the three objects represented the participant’s eye-gaze. This approach allowed for the investigation of neural differences between successful initiation of joint attention and mere gaze following. A main effect was found for joint attention resulting in the activation of the medial prefrontal cortex, posterior cingulate cortex, and the anterior temporal poles.

##### Virtual interactions using cyberball

Social neuroscientists have also started using the virtual gaming task called Cyberball to induce social exclusion in participants. A number of researchers have used the Cyberball game as an experimentally controlled social exclusion assessment that elicits affective (Williams, 2007; Wesselmann et al., 2012), neurobiological (Eisenberger, 2012), psychophysiological (Gunther Moor et al., 2010; Sijtsema et al., 2011), and hormonal (Geniole et al., 2011; Zwolinski, 2012) responses. Throughout the Cyberball task, the participant is represented by an avatar that is playing catch with two other avatars. The two other avatars ostensibly represent two other human participants. Participants are either included or ostracized during the Cyberball tossing game by two or three other players who are, in fact, controlled by an experimenter. The virtual Cyberball game starts with each avatar catching and throwing a ball (each about a third of the time). During the “inclusion” condition, the participant continues to catch and throw the ball about a third of the time. However, during the “exclusion” condition, the other two avatars throw the ball back and forth and ignore (neither avatar looks at or throws the ball to) the participant. It is interesting to note that telling participants that the avatars in the Cyberball game are controlled by a computer does not change the effects of ostracism. In fact, the ostracism delivered by computers was judged by participants to be just as unpleasant as ostracism by humans. Further, it did not matter to participants whether the human controlled or computer controlled players had a choice as to whom they threw the ball (Zadro et al., 2004).

The use of the Cyberball game in ostracism research is expedient because it allows for flexibility modification for study of group interactions without the use of live confederates, to collect large samples over the Internet, and for neuroimaging studies. Recent results from neuroimaging studies have revealed that the experience of being excluded from ball-tossing reliably evokes increased activation of the dorsal anterior cingulate and anterior insula which correlates with self-reports of physical pain (Eisenberger, 2012). A number of qualitative reviews of the fMRI and Cyberball social exclusion literature have emerged and all have concluded that nociceptive stimuli and social rejection both activate this physical pain matrix. Although the results from a recent meta-analysis suggest that the neural correlates of nociceptive stimuli and social rejection have some distinct patterns of activation, they still share commonalities (Cacioppo et al., 2013). In a more recent meta-analysis, Rotge et al. (2014) found that the Cyberball task activated the dorsal anterior cingulate circuit less than other experimental social pain tasks. These findings are consistent with the suggestion that the social pain that follows from Cyberball is less intense than the social pain that follows from more personal forms of social rejection (Eisenberger, 2015).

While the Cyberball paradigm has been widely used, some of the less robust findings may reflect the fact that early versions of the Cyberball task lacked the everyday realism and ecological validity that are now available in today’s immersive virtual environments. A recent advance in the Cyberball paradigm is an immersive virtual environment version that places the participant into a virtual environment with interactive virtual humans (Kassner et al., 2012). Results revealed that the more immersive virtual environments induced feelings of ostracism in participants. In addition to prompting feelings of ostracism that are consistent with negative effects found in minimalist environments, the immersive virtual environment effect sizes were medium to large in magnitude. In addition to these robust effects, the immersive virtual environment Cyberball paradigm offers researchers the ability to control aspects (proxemics and non-verbal communication) of the social context that cannot be accomplished in minimalist ostracism paradigms. The inclusion of immersive virtual environments in Cyberball paradigms may allow for enhanced flexibility in manipulation of social information about the confederates’ avatars, virtual humans, and/or their behaviors (Wirth et al., 2011). Further, the inclusion of virtual humans enhances the Cyberball paradigm because it allows for additional social information such as non-verbal (e.g., eye-gaze) information that has been found to convey ostracism (Wirth et al., 2010).

##### Virtual representations of self and other

Embodiment accounts of social cognition that emphasize shared bodily representations for self and other may be aided by the use of virtual reality technology. Given that immersive virtual environments involve flexible computer platforms, the shape, form, size, or type of a virtual human body can be manipulated to represent something divergent from the participant’s actual body. This flexibility of the virtual environment and the virtual human bodies can influence the participant’s perceptions, attitudes, and behaviors (Slater and Sanchez-Vives, 2014). Transcending the self in immersive virtual reality. While there have been a number of studies looking at social behaviors of participants when they are exposed to virtual humans from other demographic groups (Dotsch and Wigboldus, 2008), there is increasing interest in the use of interactive virtual humans to examine the impact of virtual embodiment on implicit social cognitions related to the other’s age (Banakou et al., 2013); body shape/size (van der Hoort et al., 2011; Normand et al., 2011; Preston and Ehrsson, 2014); gender groups (Slater et al., 2010); and race (Maister et al., 2013; Peck et al., 2013). For example, Peck et al. (2013) demonstrated that when participants experienced control of a dark-skinned virtual human there was a decrease in implicit racial bias. Likewise, in an age-related study Banakou et al. (2013) investigated the relationship between implicit attitudes and embodiment in adult participants that were immersed in virtual child bodies. Findings revealed that the adults inhabiting the virtual child’s body overestimated the size of objects. The participants also demonstrated implicit attitude and behavioral changes that appeared more child-like. It is interesting to note that when the participants were in an adult body that was the size of a child’s body they did not exhibit such changes. Maister et al. (2015) have suggested that changes in implicit attitudes occur via a process of self-association that occurs in the physical feeling (i.e., perceived increase in bodily similarity between self and other) domain and then extends to the cognitive (conceptual generalization of positive self-like associations to the other) domain.

## Summary and Discussion

The purpose of the review is to emphasize the potential of virtual reality for precise presentation and control of dynamic perceptual stimuli that can be used for assessment of neurocognitive and affective processing while participants are immersed in simulations of real world contexts. Historically, this work has relied on simple and static stimuli (e.g., Stroop for clinical; IGT for affective, and schematic faces for social neuroscience) lacking many of the potentially important advantages in advanced technology and characteristics of real world activities and interactions. The current review suggests that the promise of the approach described here has already started to be realized. For example, in the clinical neurosciences virtual environment-based neuropsychological assessments allow for real-time assessment of a participants cognitive and affective processing in a manner that more closely resemble real-world functional abilities (Matheis et al., 2007; Parsons and Rizzo, 2008). In affective neuroscience, virtual environments are also being used to assess “Hot” processes of affective arousal both clinical (Parsons and Rizzo, 2008) and nonclinical (Armstrong et al., 2013; Parsons et al., 2013) studies. Finally, for social neuroscience, virtual environments offer the social neuroscientist the ability to induce a feeling of “presence” in participants as they experience emotionally engaging background narratives to enhance affective experience and social interactions (Gorini et al., 2011; Diemer et al., 2015).

It is important to reiterate that the use of virtual environments advocated here does not seek to minimize the contribution of traditional (paper-and-pencil and computerized) neuropsychological assessments that use static stimuli. The neuropsychological assessment measures in clinical neuroscience and the static stimuli in social neuroscience have numerous benefits for researchers as evidenced by the progress made using such tests and stimuli. The approach advocated here calls for the “addition” (not replacement) of virtual environments to neuropsychological batteries in situations where researchers desire to have some idea of real world functioning. Another important note is that in some neuroimaging research, the approach suggested will present methodological challenges. While this is a challenge, there is no reason to abandon such an effort. Understanding the neural correlates of Cold and Hot processing using virtual environments represents an important advance in clinical, affective, and social neuroscience. This pursuit faces challenges given the complex nature of real world interactions. This review presents one viable way to meet some of the challenges. Given that virtual environments allow for precise presentation and control of dynamic perceptual stimuli, they can provide ecologically valid assessments that combine the control and rigor of laboratory measures with emotionally engaging background narratives to enhance assessment of cognition, affect, and social interactions.

## Conflict of Interest Statement

The author declares that the research was conducted in the absence of any commercial or financial relationships that could be construed as a potential conflict of interest.
